# Length-of-stay after a health facility birth and associated factors: analysis of data from three Sub-Saharan African countries

**DOI:** 10.4314/gmj.v56i2.7

**Published:** 2022-06

**Authors:** Fatimah I Tsiga-Ahmed, Rabiu I Jalo, Usman M Ibrahim, Aminatu A Kwaku, Amina A Umar, Surayya M Sanusi, Taiwo G Amole

**Affiliations:** 1 Department of Community Medicine, College of Health Sciences, Bayero University Kano, Nigeria; 2 Department of Community Medicine, Aminu Kano Teaching Hospital, Kano, Nigeria; 3 African Centre of Excellence for Population Health and Policy, Bayero University Kano, Nigeria

**Keywords:** length-of-stay, childbirth, health facility, Africa

## Abstract

**Objectives:**

We estimated the length-of-stay (LOS) in the health facility after childbirth and identified associated factors in three sub-Saharan African countries.

**Design:**

Secondary analysis using data from the most recent Multiple Indicator Cluster Surveys.

**Setting:**

Multiple Indicator Cluster Surveys from Ghana, Malawi and Eswatini were selected.

**Participants:**

Women aged 15–49 years who had a facility delivery in the two years preceding the survey were included.

**Main outcome measures:**

Length-of-stay recorded in days and weeks were converted to hours and analysed as a continuous variable.

**Results:**

Length-of-stay was estimated for 9147 women, wherein 6610 women (median LOS and IQR: 36 36,60 hours), 1698 women (median LOS and IQR 36 10,60 hours) and 839 women (median-length-stay 36 36,60 hours) were from Malawi, Ghana and Eswatini respectively. Being from Ghana [RC, -20.6 (95%CI:-25.2 - -16.0)] and then Eswatini [RC: -13.0 (95%CI: -19.9 - -9.8)] and delivery in a government hospital [RC: -4.9 (95%CI -9.9- -0.3)] were independently associated with having a shorter LOS. Having a caesarean section, assistance by Nurses/Midwives or Auxiliaries/CHOs, single birth, heavier birth weight, and death of newborn before discharge increased the duration of stay.

**Conclusions:**

Necessitating and facility factors are important determinants of length of stay. Socio-demographic characteristics, however, have a restricted role in influencing the duration of postpartum stay in sub-Saharan Africa. Further prospective research is required to identify more determinants and provide evidence for policy formulation and clinical guidelines regarding the safest time for discharge after delivery.

**Funding:**

None declared

## Introduction

The global burden of maternal and perinatal death is enormous despite being a global public health priority.^1^, ^2^ Risk of death in most settings is highest during the intra-partum and early postpartum period, with 45%–50% of maternal deaths and 24%–45% of neonatal deaths occurring within this phase.^3^–^6^ To reduce these figures, the World Health Organization (WHO) recommended that deliveries occur at facilities where skilled birth attend-ants' supervision is possible.^7^ The fundamental theory underlying this decision is that unpredictable and fatal complications could be detected and managed early.

Monitoring women and their newborn babies for an adequate time is essential, given that certification of stability in the mother-baby dyad before discharge is only possible if an observation is carried out over a sufficient period.

Opinions on what defines untimely discharge are diver-gent across experts, and recommendations for appropriate LOS have ranged from 12 to 96 hours for uncomplicated births.^8^–^12^ Although the WHO has endorsed a stay of at least 24 hours in the facility after an uncomplicated delivery,^13^ there is an increasing trend in early discharge, with some countries making policy changes to curtail that action.^14^ A study seeking to describe the LOS after facility delivery in 92 countries discovered that between 0.2% to 83% of women who had vaginal deliveries stayed for less than 24 hours and between 1% to 75% of women who had caesarean-section (CS) deliveries stayed for less than 72 hours.^15^

Sub-Saharan Africa contributes more than half of all maternal and newborn deaths in low-income settings.^5^ Ghana with a maternal mortality ratio (MMR) of 380 per 100,000 live births, Malawi with an MMR of 574/100,000 and Eswatini with an MMR of 589/100,000 are countries with high mortality rates. Between 11.1% to 27.9% of births occur outside a hospital in Ghana, Malawi and Eswatini.^16^–^18^ Consequently, for the women who make it to the facilities, it seems prudent to keep them for an adequate duration to ensure they get appropriate care before discharge. Unfortunately, few studies on LOS from many low-income settings like sub-Saharan Africa exist.

Length-of-stay has been used to evaluate perinatal policies,^19^ and health professionals and policymakers have raised concerns regarding the impact of reduced LOS on maternal and child morbidity. Early discharge, if accompanied by a home visit, has been associated with improved maternal emotional and physical health, decreased hospital-acquired infections, and reduced financial burden on the individuals and the health system.^9^, ^20^–^27^. Nonetheless, insufficient LOS is responsible for a reduction in the quality of medical assistance received after birth, increased risk of misdiagnosis, breastfeeding problems, increased neonatal readmission, dissatisfaction with health services and increased risk of readmission in women who had caesarean births.^5^, ^9^, ^28^–^40^

Although the most mentioned factors associated with LOS after delivery appear similar, continental variabilities prevail. In high-income settings, differential lengths of stay have been due to an overlap between institutional and socio-demographic factors.^41^ Sub-Saharan Africa being chiefly low-income, may have different determinants. A study in Tanzania discovered an indistinct relationship between LOS and many socio-demographic characteristics contrary to what obtains elsewhere.^5^ Therefore, there is a compelling need to assess the LOS after birth to identify settings where women do not benefit fully from having a facility delivery. This paper seeks to determine LOS and identify associated factors in three sub-Saharan African countries.

## Methods

Data from Multiple Indicator Cluster Surveys (MICS) conducted in Ghana, Malawi and Eswatini were analysed. MICS is an international household survey developed and supported by the United Nations Children's Fund (UNICEF) to produce data on household, maternal, and child health indicators.

It provides comprehensive and globally comparable data collected from an average of 11,000 households. Information on socio-demographic variables, place of delivery, LOS after delivery, newborn characteristics and specific behaviours of women that may influence LOS after childbirth are provided. This study used data from the Malawi 2014 MDG Endline Survey, Ghana 2011 MICS and Eswatini 2014 MICS.

### Study population

Consenting women aged 15 to 49 who were de jure members of sampled households in all three countries comprised the study population.

### Length-of-stay

Multiple Indicator Cluster Surveys collected data from women who had given birth in the two years preceding the survey. Women were asked whether they delivered in a facility and their length of stay in the facility after giving birth. Length-of-stay was recorded in hours if less than a day, days if less than one week, and weeks if up to a week or more. LOS (in hours) was the outcome of interest and was expressed as a continuous variable. For LOS originally recorded in hours, various responses of less than 24 hours were seen and thus, the presumption that a day was 24 hours and more. To account for the range of hours in days, the midpoint of recorded days was used. For example, a day could range from 24 to 47 hours. Thus, the midpoint of 36 hours was used for one day, 60 hours for two days and similar assumptions for other days.

Duration of stay recorded in weeks was also changed to mid-points of the weeks and converted to hours. In this instance, one week, which could range between 7 and 13 days, was converted to 10.5 days, and two weeks with a range of 14 to 20 days was converted to 17.5 days. Delivery at a mission hospital was only available for Malawi. To maintain uniformity, all deliveries in the missionary hospital in Malawi were considered as being in a private hospital.

### Statistical analyses

All analyses were done using STATA version 14.2 (Stata Corp, College Station, TX, USA). Data was not self-weighting; to account for survey design (disproportionate stratification), calculated individual women survey weights were applied to all analyses. Frequencies and percentages of women who had a facility delivery and their characteristics were estimated and described. Weighted medians with interquartile range (IQR) of LOS for various factors were calculated.

Linear regression techniques were used for univariate and multivariable analysis of the relationship between factors and LOS.

Verification of normality assumption revealed that LOS from this data was slightly skewed to the right, with apparent influential points. Scrutiny of unanalysed data revealed that this was most likely from data reporting or entry errors. Including these values in regression models could result in incorrect coefficients and distorted distribution on a linear scale. To arrive at appropriate inferences, a decision was made to use an upper limit of three weeks duration for extreme values and exclude outliers beyond this (1.0%).

Crude associations between LOS and each of the exposures of interest were explored. The parameter of interest was the regression coefficient of expected increase (or decrease) in LOS expressed in hours, along with its 95% confidence interval. Evidence of a difference in the coefficients between categories of explanatory variables was obtained from a Wald test. Mode of delivery and country were regarded as a priori confounders. Subsequently, country-adjusted analysis and country/mode of delivery-adjusted associations were examined to obtain partially-adjusted (country-adjusted and country/mode of delivery-adjusted) regression coefficients for each variable.

Multivariable linear regression was employed to assess the independent effects of all variables on LOS. A forward approach to modelling was adopted, starting with a priori confounders in the base model and sequentially adding variables with a p-value ≤0.10. A final model was built by excluding the variables individually until all remaining factors had a p-value of <0.05. Partial-F tests were used to simultaneously evaluate the impact of several parameters and obtain evidence of associations.

A log-transformation of LOS was done, and linear regression was repeated; however, regression coefficients obtained were similar to those obtained from initial models using untransformed data. Therefore, results from previous untransformed data were presented for easier understanding and interpretation.

### Ethical Considerations

Ethical approval for analysis of this data was obtained from the London School of Hygiene and Tropical Medicine (LSHTM) Research Ethics Committee (Ref – 13658).

UNICEF and National Statistical Offices of Ghana, Malawi and Eswatini obtained ethical clearance and permission from appropriate ethics review boards in the respective countries. Permission to use the data was sought from UNICEF.

## Results

The original dataset contained information on 10,977 (weighted) women who had a live birth. Among these, 9206 were delivered in the hospital, of which 9147 (99.4%) were found to have relevant information, hence eligible for analysis. Out of these, 6610 (72.3%), 1698 (18.5%) and 839 (9.2%) were from Malawi, Ghana and Eswatini, respectively. About a fifth (21.8%) lived in urban areas, with 2191(24.0%) being educated up to secondary level and approximately half (n=4792, 52.4%) in their third decade of life. Further description of the study participants is in Table 1.

**Table 1:** Distribution of explanatory variables among participants and median length-of-stay in hours by country and mode of delivery

Factor (Number of Missing Observations)	Number of Participants^1^				Median LOS in hours (Interquartile range)			
	All countries (%) N=9147	Malawi (%) n=6610	Ghana (%) n=1698	Eswatini (%) n=839	All countries n=9147^1^	Malawi n=6610^1^	Ghana n=1698^1^	Eswatini n=839^1^
**Overall Median LOS**					36(36,60)	36(36,60)	36(10,60)	36(36,60)
**Mode of delivery (1)**								
**Vaginal**	8374(91.6)	6230(94.3)	1414(83.3)	729(86.9)	36(36,60)	36(36, 60)	36(9, 60)	36(36, 36)
**Caesarean section**	772(8.4)	378(5.7)	284(16.7)	110(13.1)	132(84,252)	180(84,252)	108(84,156)	84(84,132)
**Residence**								
**Urban**	1998(21.8)	828(12.5)	931(54.9)	239(28.5)	36(36,60)	36(36,60)	36(12,84)	36(36,60)
**Rural**	7149(78.2)	5783(87.5)	767(45.1)	600(71.5)	36(36,60)	36(36,60)	36(9,60)	36(36,60)
**Women's age in years (0)**								
**15–19**	1147(12.5)	924(14.0)	108(6.4)	115(13.6)	36(36,60)	36(36,60)	36(10,60)	36(36,60)
**20–24**	2452(26.8)	1883(28.5)	313(18.4)	255(30.4)	36(36,60)	36(36,60)	36(11,60)	36(36,60)
**25–29**	2340(25.6)	1678(25.4)	446(26.3)	217(25.8)	36(36,60)	36(36,60)	36(13,84)	36(19,60)
**30–34**	1786(19.5)	1211(18.3)	434(25.6)	141(16.8)	36(36,60)	36(36,60)	36(10,84)	36(36,84)
**35–39**	97(10.7)	638(9.6)	265(15.6)	74(8.8)	36(36,60)	36(36,60)	36(9,84)	36(36,60)
**40–44**	35(3.9)	226(3.4)	98(5.8)	34(4.0)	36(36,60)	36(36,60)	36(10,60)	60(36,84)
**45–49**	89(1.0)	51(0.8)	33(2.0)	5(0.1)	36(36,60)	36(36,60)	36(14,60)	60(60,84)
**Education (1)**								
**None**	1040(11.3)	710(10.7)	309(18.2)	22(2.6)	36(36,60)	36(36,60)	36(36,60)	36(36,60)
**Primary**	5915(64.7)	4655(70.4)	1064(62.7)	196(23.4)	36(36,60)	36(36,60)	36(10,60)	36(36,60)
**Secondary and higher**	2191(24.0)	1245(18.8)	325(19.1)	621(74.0)	36(36,84)	36(16,60)	36(18,84)	36(36,60)
**Marital status (2)**								
**Currently married**	7536(82.4)	5596(86.7)	1508(88.8)	432(51.5)	36(36,60)	36 (36,60)	36(10,60)	36(36,60)
**Formerly married**	864(9.5)	751(11.4)	54(3.2)	60(7.1)	36(36,60)	36 (36,60)	17(9,60)	36(36,60)
**Never married**	747(8.1)	261(4.0)	136(8.0)	347(41.4)	36(36,60)	36 (36,84)	36(13,60)	36(36,60)
**Wealth index quintile (0)**								
**Poorest**	1930(21.1)	1564(23.7)	211(12.4)	155(18.5)	36(36,60)	36(36,60)	36(8,60)	36(36,60)
**Second**	1943(22.2)	1451(21.9)	310(18.2)	183(21.8)	36(36,60)	36(36,60)	36(8,60)	36(36,60)
**Middle**	1889(20.7)	1368(20.7)	343(20.2)	178(21.2)	36(36,60)	36(36,60)	36(14,60)	36(36,60)
**Fourth**	1666(18.2)	1119(16.9)	383(22.6)	164(19.5)	36(36,60)	36(36,60)	36(11,60)	36(36,60)
**Richest**	1719(18.8)	1109(16.8)	452(26.6)	159(18.9)	36(36,60)	36(36,60)	36(13,84)	36(36,60)
**Wanted child at time of delivery (2)**								
**Yes**	4990(54.6)	3690(55.8)	967(57.0)	333(39.7)	36(36,60)	36(36,60)	36(10,60)	36(36,60)
**No**	4155(45.4)	2918(44.2)	731(43.0)	506(60.3)	36(36,60)	36(36,60)	36(11,60)	36(36,60)
**Birth order (0)**								
**1**	2403(26.3)	1628(24.6)	474(27.9)	300(35.7)	36(36,60)	36(36,60)	36(14,84)	36(36,60)
**2–3**	3423(37.4)	2425(36.7)	634(37.3)	364(43.4)	36(36,60)	36(36,60)	36(10,60)	36(36,60)
**4–6**	2587(28.3)	1969(29.8)	465(27.4)	153(18.3)	36(36,60)	36(36,60)	36(8,60)	36(36,60)
**7+**	734(8.0)	588(8.9)	124(7.3)	22(2.6)	36(36,60)	36(36,60)	36(13,84)	36(36,60)
**Type of delivery (0)**								
**Singleton**	8956(97.9)	6471(97.9)	1658(97.6)	827(98.6	36(36,60)	36(36,60)	36(10,60)	36(36,60)
**Multiple**	191(2.1)	139(2.1)	40 (2.4)	12(1.4)	60(36,84)	60(36,84)	36(36,132)	60(36,132)
**Child's birthweight in Kg (230)**								
**<2.5**	552(6.4)	386(6.1)	147(10.3)	19(22.9)	36(36,60)	36(36,60)	36(20,84)	36(36,84)
**2.5–3.5**	1129(13.1)	876(13.7)	176(12.3)	78(9.3)	36(36,60)	36(36,60)	36(10,60)	36(36,60)
**>3.5**	6954(80.5)	5118(80.2)	1101(77.3)	734(88.4)	36(36,60)	36(36,60)	36(14,60)	36(36,60)
**Survival index of newborn (0)**								
**Survived**	9068(99.1)	6548(99.1)	1690(99.5)	830(98.9)	36(36,60)	36(36,60)	36(10,60)	36(10,60)
**Died before/on day of discharge**	17(0.2)	14(0.2)	1(0.1)	3(0.3)	36(36,60)	36(36,60)	12(5,36)	84(36,84)
**Died after discharge**	62(0.7)	49(0.7)	7(0.4)	6(0.8)	60(36,60)	60(36,132)	60(36,108)	36(36,36)
**Type of facility (0)**								
**Government**	7679(84.0)	5683(86.0)	1429(84.2)	568(67.7)	36(36,60)	36(36,60)	36(11,60)	36(36,60)
**Private**	1468(16.0)	927(14.0)	269(15.8)	271(32.3)	36(36,84)	60(36,84)	36(10,84)	36(18,60)
**Assistant during delivery (0)**								
**Doctor**	1543(16.9)	1048(15.9)	324(19.1)	172(20.5)	60(36,108)	60(36,84)	84(35,156)	60(36,108)
**Nurse/Midwife**	7277(79.6)	5276(79.8)	1334(78.6)	667(79.4)	36(36,60)	36(36,60)	36(9,60)	36(36,36)
**Auxiliary/CHO/Other**	327(3.6)	286(4.3)	39(2.3)	1(0.1)	36(36,60)	36(36,60)	13(8,36)	21(21,21)

1 Weighted based on individual women survey weights

### Length-of-stay after childbirth

**Figure 1:**
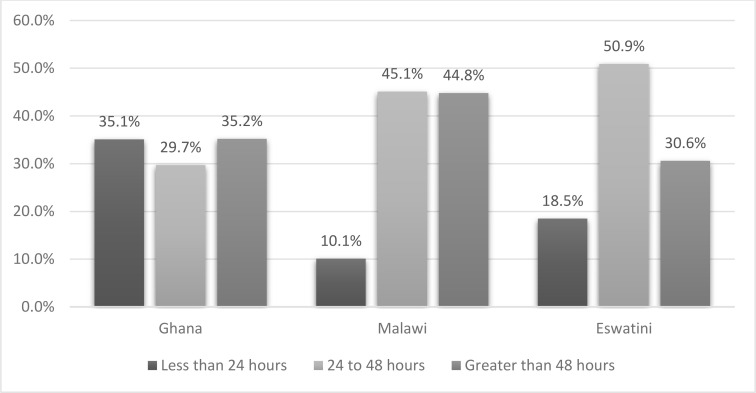
Distribution of categories of length-of-stay by country.

**Figure 2:**
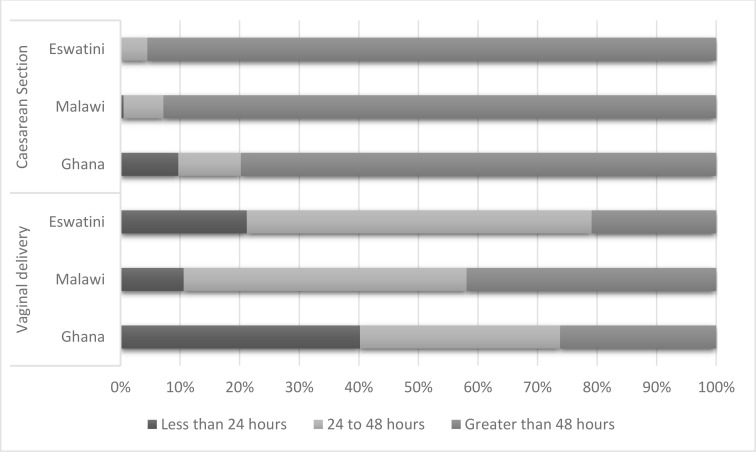
Distribution of categories of length-of-stay by mode of delivery and country

Across all countries, 1415 (15.5%) stayed in the health facility for less than 24 hours postpartum, irrespective of the mode of delivery. The highest proportion of women who stayed less than 24 hours was from Ghana (n=596, 35.1%), as shown in Figure 1. Figure 2 shows that among women who had a vaginal delivery, 1386 (16.6%) of the stayed for less than 24 hours, which was also more prevalent in Ghana (40.2%).

Table 1 shows the median LOS across all countries and by mode of delivery. Median LOS was similar across all three countries –36 hours (Malawi IQR 36,60, Ghana IQR 9-60 and Eswatini IQR 36,60). However, the median LOS varied across different assistants at delivery; LOS was highest in deliveries assisted by doctors (60 hours, IQR 36,60) compared to those assisted by nurses/mid-wives and auxiliaries/CHOs (36 hours IQR 36,60). Among all participants, median LOS differed according to the survival index of the child and gestation number. Women whose children died after discharge had a higher median LOS (60 hours IQR 36,60), and women who had multiple gestations (60 hours IQR 36,84) had higher median LOS than those who had singletons (36 hours IQR 36,60).

Upon stratification by mode of delivery, among women who delivered via caesarean section, Eswatini had the shortest LOS with a median of 84 hours IQR 83,32 relative to Ghana, which had 108 IQR 84,156 and Malawi, which had the highest median of 180 IQR 84,32 hours. Overall, the median LOS among women with CS in all three countries was132 IQR 84,252, while those who delivered vaginally had a median of 36 IQR 36,60 hours.

### Factors associated Length-of-stay after childbirth

Table 2 shows the country/mode of delivery-adjusted and the final adjusted estimates for the association between explanatory factors and LOS. The results showed that being from Ghana (and then Eswatini), having a vaginal delivery, delivery in a government hospital, assistance by nurses/midwives or auxiliaries/CHOs, single birth, heavier birth-weight and death of newborn before discharge were independently associated with having a shorter LOS. Compared to Malawi, women from Ghana and Eswatini had shorter LOS (p≤0.001), [Ghana RC:-20.6 (95%CI:-25.2- -16.0)], [Eswatini RC:-13.0 (95%CI: -19.9- -9.8)]. Additionally, women who delivered in government hospitals had shorter LOS than those who gave birth in private hospitals [RC: -4.9 (95%CI:-9.9–0.3)]. Similarly, those whose delivery was assisted by nurses/midwives and auxiliaries/community health officers had shorter lengths of stay than those assisted by a doctor (p≤0.001).

**Table 2:** Factors associated with Length-of-stay after facility birth

Factor	p-value^2^	Regression coefficient Adjusted for country and mode of delivery	p-value*	Fully adjusted model Ɨ	p-value
**Country**					
**Malawi**	<0.001	Reference	<0.001	Reference	<0.001
**Ghana**		-19.5 (-24.4 – -14.5)		-20.6 (-25.2 – -16.0)	
**Eswatini**		-17.3 (-21.8 – -12.9)		-14.8 (-19.9 – -9.8)	
**Residence**					
**Urban**	0.76	Reference	0.76		
**Rural**		0.8 (-4.1 – 5.6)			
**Women's age in years**					
**15–19**	0.16	Reference	0.20		
**20–24**		1.5 (-3.8 – 6.9)			
**25–29**		3.4 (-2.7 – 9.5)			
**30–34**		-1.7 (-7.3 – 3.9)			
**35–39**		0.3 (-7.0 – 7.7)			
**40–44**		4.8 (-3.9 – 13.4)			
**45–49**		16.5 (-5.6 – 38.5)			
**Educational status**					
**None**	0.10	Reference	0.56		
**Primary**		-3.3 (-9.9 – 3.3)			
**Secondary and higher**		-4.1 (-11.5 – 3.4)			
**Marital Status**					
**Currently married**	0.21	Reference	0.42		
**Formerly married**		4.1 (-2.3 – 10.2)			
**Never married**		-1.4 (-8.1 – 5.4)			
**Wealth Index Quintile**					
**Poorest**	0.38	Reference	0.53		
**Second**		0.8 (-4.4 – 6.1)			
**Middle**		-2.1 (-7.0 – 2.7)			
**Fourth**		0.6 (-4.4 – 5.6)			
**Richest**		-2.8 (-8.4 – 2.7)			
**Other children at home**					
**Yes**	0.07	Reference	0.02	Reference	0.24
**No**		17.8 (2.6 – 33.1)		5.4 (-6.5 – 17.4)	
**Attendance of ANC**					
**Yes**	0.87	Reference	0.58		
**No**		-3.9 (-17.9 – 10.1)			
**Wanted child at the time of delivery**					
**Yes**	0.05	Reference	0.15	Reference	0.19
**No**		-2.3 (-5.5 – -0.8)		-2.1 (-5.3 – 1.1)	
**Sex of child**					
**Male**	0.65	Reference	0.90		
**Female**		0.9 (-3.0 – 3.5)			
**Birth order**					
**1**	0.007	Reference	0.62		
**2–3**		-0.8 (-5.0 – 3.2)			
**4–6**		-2.6 (-6.6 – 1.4)			
**≥7**		-2.0 (-7.7 – -3.7)			
**Type of facility**					
**Private**	0.03	Reference	0.006	Reference	0.006
**Government**		-4.1 (-8.6 – 0.44)		-4.9 (-9.5– -0.3 )	
**Assistance during delivery**					
**Doctor**	<0.001	Reference	<0.001	Reference	<0.001
**Nurse/Midwife**		-11.6 (-17.2 – -6.0)		-10.5 (-16.1 – 4.9)	
**Auxiliary/CHO/Others**		-16.2 (-27.1 – -5.4)		-17.9 (-25.4 – -10.3)	
**Mode of delivery**					
**Vaginal**	<0.001	Reference	<0.001	Reference	<0.001
**Caesarean section**		113.4 (102.9 – 123.9)		104.0 (92.5 – 113.8)	
**Gestation number**					
**Singleton**	<0.001	Reference	<0.001	Reference	<0.001
**ultiple**		43.4 (21.4 – 65.3)		40.9 (17.4 -64.3)	
**Baby's birthweight in kg**					
**<2.5**	<0.001	Reference	<0.001	Reference	<0.001
**2.5–3.5**		21.0 (-29.8 – -12.3)		-18.4 (-26.7 – -9.9)	
**>3.5**		-21.3 (-30.2 – -12.5)		-18.3 (-26.9 – -9.7)	
**Survival of child**					
**Survived**	<0.001	Reference	0.02	Reference	0.007
**Died before/on day of discharge**		-6.2 (-21.2 – 8.8)		-19.0 (-38.7 – 0.7)	
**Died after discharge**		40.5 (8.3 – 72.7)		20.7 (12.4 – 53.8)	

2 *p*-values from Wald test

* *p*-values from partial F test

Ɣ Adjusted for all covariates in the model

Analyses also revealed that the mode of delivery was strongly associated with LOS (p≤ 0.001). Women who delivered via CS had 104 additional hours compared to those who had a vaginal delivery [RC:104.0 (95%CI: 92.5–113.8)]. Furthermore, women with twins or more had longer LOS than those with singleton [RC:40.9 (95%CI: 17.4–65.3)]. Having a baby with a birth weight greater than 2.5kg was independently associated with a shorter LOS [RC:18.4 (95%CI -26.7- -9.9) among newborns weighing 2.5–3.5kg and RC:18.3(95%CI -26.9 — -9.7) among those greater than 3.5kg]. Also, women whose children died after discharge had much longer stay compared to those whose children survived [RC20.7(95%CI -12.4 — 53.8], while those whose children died on or before the day of discharge had shorter LOS [RC -19.0 (95%CI: -38.7–0.7)].

## Discussion

Based on the WHO recommendation on acceptable LOS after childbirth,^13^ the results of this study show that a sizeable number of women were discharged early in these populations. The results of the multivariable model showed that being from Eswatini (and then Ghana), having a vaginal delivery, delivery in a government hospital, assistance by auxiliaries/CHOs, single birth, heavier birth-weight and death of newborn on or before the day of discharge were independently associated with having a shorter LOS.

In this study, a tenth to almost half of women with vaginal births stayed for less than the recommended 24 hours. In Africa, discharge within 24 hours of a facility delivery is considered an institutional norm and a societal expectation except in the presence of a complication or caesarean section.^5^ A study in Tanzania found that although time of discharge was decided by healthcare providers, women were happy to leave before 24 hours either due to a longing to be home or due to some facility-related discontentment.^5^ Similar to our findings, their study revealed that early discharge was usual in their setting, with 65.7 % of uncomplicated vaginal deliveries routinely discharged within 12 hours and 90% within 24 hours. Additionally, the study showed that about 10.5% of women who had a CS and 44.2% of those who had a complication also left before 24 hours. Our findings show variations in LOS between the three countries, with Ghana having the highest proportion of women discharged before 24 hours. This finding may be attributed to a high proportion of singleton births, deliveries in a government facility and a high newborn survival rate among participants from this country. These factors have been associated with shorter LOS in our study.

Having a CS was independently and consistently associated with longer LOS. A study reported that the more complicated mode of deliveries were expected to be associated with extended hospital stays.^41^

Women who have caesarean deliveries, irrespective of demographic and obstetric characteristics, have a higher risk of maternal morbidity compared to vaginal deliveries,^42^–^47^ and this could increase the duration of hospitalization. Another study from South Africa found that 62.5 % of women who delivered by CS experienced a severe maternal outcome compared to 37.5% of vaginal deliveries.^46^ Both the underlying indication for CS and consequences of CS like hysterectomy, blood transfusion, surgical infection and anaesthesia-related problems could increase the LOS after childbirth.

This study found no association between LOS and socio-demographic factors after adjusting for variables in the final model. Diverse outcomes on the relationship between socio-demographic characteristics and LOS have been obtained across different populations.^5^, ^48^,^49^–^54^ Results obtained from similar settings found no association between LOS and demographic characteristics.^5^ This study highlighted that the responsibility of discharge in sub-Saharan Africa was chiefly vested on caregivers. Therefore, individual or interpersonal characteristics of women had a limited role in deciding the duration of stay. Indeed, women stayed for a duration the provider felt was adequate.

Our findings highlight that women delivering in private hospitals consistently had a longer LOS than those in public hospitals. These findings might be due to a difference in the technical performance of these hospitals or who self-selects to attend them. Public hospitals are more overwhelmed, and perhaps women are discharged early so others can use the bed space. Deliveries attended by non-doctors had shorter lengths-of-stay across all countries and all modes of delivery. Sub-Saharan Africa has been experiencing a critical shortage of human resources, with 31 countries falling below the critical threshold of 2.3 per 1000 population.^55^, ^56^ This scarcity is adversely affecting the availability of services and quality of care given, especially among the lower cadre of health workers. Non-doctors attend to the majority of uncomplicated deliveries and the doctors attend to the complicated ones, which may require longer duration of admission.

Women who had multiple gestation or infants with low birth-weight stayed longer. Both birthweight and multiple gestations have been identified as significant determinants of maternal and neonatal morbidity.^57^–^59^ Babies with low birth weight are at higher risk of complications and would require longer lengths of admission. Caesarean sections, which increase the LOS, are also higher among multiple gestation births.^60^ Women whose newborns died on/before discharge consistently had shorter lengths-of-stay. This is not unusual; similar to stillbirth, women usually require family support and want to be quickly away from the environment, reminding them of the horrible ordeal. In this study, 34.6% of these deaths occurred on the day of birth compared to none among mothers whose children died after discharge. A study has shown that women with stillbirth had a significantly shorter stay than those with live babies.^61^

Additionally, a shorter LOS among this group could be because survival is a proxy for the need for care. Newborns who survive complications could be on admission for a more extended period, whereas those with severe complications die within a short time. Overall results of this study are in keeping with a much larger study that looked at similar data, where factors relating to need and health system had the strongest relationship with LOS but was unable to demonstrate the association they found between socio-demographic variables and LOS.^48^

A strong point of our study is the large sample size with a high response rate. Data were merged from three countries to obtain a large sample size with sufficient power to detect the most negligible differences between groups. Nevertheless, our findings should be cautiously interpreted considering that the study only included women who were alive and excluded stillbirths. Misclassification of outcome and exposure variables from recall or interviewer bias is a possibility although, very likely to be non-differential. The high proportion of non-doctor attended deliveries among women who had CS could also be due to reporting errors, either because women do not know which cadre attended them or because they are not clear on what caesarean-section is as discovered by a recent study.^62^

Although across-the-board conclusions cannot be drawn due to the self-report and secondary nature of this data, it is proposed that LOS be viewed as a highly relevant issue requiring attention and investigation, especially in sub-Saharan Africa. Exploration of isolated country-specific determinants is suggested.

## Conclusion

Across all three countries, almost 1 in 5 women stayed for less than 24 hours after a facility delivery, with Ghana having the highest proportion of women in this category. However, among women who had a vaginal delivery between a tenth to almost half stayed for less than the recommended 24 hours. Length of stay was influenced by country, mode of delivery, type of facility where delivery was made, cadre of assistant at delivery, baby's birth weight, gestation number and survival of child. Leveraging on the fact that gestational, facility and necessitating factors were the key determinants of LOS, we recommend investments in human resources and facility infra-structure to reduce hospital congestion and discomfort, improve nursing support, increase awareness and allow for home visits during early postpartum period.
